# Is carbonic anhydrase inhibition useful as a complementary therapy of Covid-19 infection?

**DOI:** 10.1080/14756366.2021.1924165

**Published:** 2021-06-02

**Authors:** Secil Deniz, Tugba Kevser Uysal, Clemente Capasso, Claudiu T. Supuran, Ozen Ozensoy Guler

**Affiliations:** a Department of Infectious Diseases, Faculty of Medicine, Pamukkale University, Denizli, Turkey; b Department of Medical Biology, Faculty of Medicine, Ankara Yildirim Beyazit University, Ankara, Turkey; c CNR-Institute of Biosciences and Bioresources, Napoli, Italy; d NEUROFARBA Department, Section of Pharmaceutical and Nutriceutical Chemistry, Università degli Studi di Firenze, Laboratorio di Chimica Bioinorganica, Florence, Italy

**Keywords:** Covid-19, CO_2_, hypoxia, carbonic anhydrase, high-altitude pulmonary oedema

## Abstract

The ongoing Covid-19 is a contagious disease, and it is characterised by different symptoms such as fever, cough, and shortness of breath. Rising concerns about Covid-19 have severely affected the healthcare system in all countries as the Covid-19 outbreak has developed at a rapid rate all around the globe. Intriguing, a clinically used drug, acetazolamide (a specific inhibitor of carbonic anhydrase, CA, EC 4.2.1.1), is used to treat high-altitude pulmonary oedema (HAPE), showing a high degree of clinical similarities with the pulmonary disease caused by Covid-19. In this context, this preliminary study aims to provide insights into some factors affecting the Covid-19 patients, such as hypoxaemia, hypoxia as well as the blood CA activity. We hypothesise that patients with Covid-19 problems could show a dysregulated acid–base status influenced by CA activity. These preliminary results suggest that the use of CA inhibitors as a pharmacological treatment for Covid-19 may be beneficial.

## 1. Introduction

World Health Organization (WHO) presents official daily counts of Covid-19 cases and deaths worldwide. On 15 March 2021, WHO declared 119,603,761 confirmed cases of COVID-19, including 2,649,722 deaths. Coronaviruses (CoVs) are a family of viruses (Coronaviridae) that cause the common cold or more severe diseases, such as the Middle East Respiratory Syndrome (MERS) and the Severe Acute Respiratory Syndrome (SARS). The term "coronavirus" maybe was unknown to many, but most of us have encountered milder forms of these viruses. Four of these viral strains cause about one-fifth of all common cold cases, while other types cause endemic diseases in some animal populations. As all known human CoV strains caused relatively mild infections, research in this area lags behind other viruses that caused worse diseases (e.g. HIV, HCV, herpes viruses, etc.). Everything changed in 2003 when the CoV was identified as responsible for the SARS outbreak in China. This virus passed from animals, most likely civets, to humans (zoonosis). The virus propensity to make the spill-over became evident in 2012, when another virus passed from camels to humans, causing MERS, which killed 858 people (about 34% of those infected), mainly in Saudi Arabia. Near the end of 2019, a novel virus named SARS-CoV-2, previously not identified in humans, caused the outbreak of a new disease (nowadays known as Covid-19) that started in China and was rapidly spread in Asia, Europe, the USA, Australia, and the rest of the world. It had been at least 100 years since humankind had been confronted with such a pandemic, whose severity has been attributed to WHO delay in recognising the virus responsible for Covid-19^1^. Like SARS and MERS, Covid-19 is a zoonosis in which bats are probably the source of the virus, and other mammals were intermediary hosts that, subsequently, can infect humans. The recent SARS-CoV-2 genome analysis revealed that it shares 96% of its RNA with a previously identified CoV in a bat species from China. There were no bats for sale at the animal market of Wuhan (China), where the current outbreak has started, suggesting that another intermediate host species was probably involved, which seems to be the pangolin^2^. SARS, MERS, and Covid-19 share a common feature: intermediate hosts that increase the genetic diversity of viruses by facilitating more or different mutations, and therefore, affording the spill-over of the virus to humans^2^. The common symptoms provoked by SARS-CoV-2 are fever, myalgia, dry cough, headache, sore throat, and chest pain, and therefore, it is considered a respiratory disease^3^. Angiotensin-converting enzyme 2 (ACE2), the functional receptor of SARSCoV2, plays a crucial role in the pathogenesis of Covid-19, as it provides viral entry into human cells^4^. ACE2 is a transmembrane protein located on the cells of the respiratory tract, lung, heart, arteries, veins, kidney, and intestines. In the pulmonary epithelium, ACE2 acts as a vasodepressor, balancing its counterpart the homologous enzyme ACE1, which acts as a vasoconstrictor.

### 1.1. Carbonic anhydrase (CA) and its putative role in Covid-19

CAs efficiently catalyse the reversible hydration of carbon dioxide to bicarbonate and H^+^ ions and play a crucial role in regulating many physiological processes. These enzymes are actively involved in catalysing the reversible conversion of carbon dioxide and water into a bicarbonate ion and a proton (CO_2_+H_2_O ⇆ HCO_3_^−^+H^+^) by ensuring a two‐step reaction process. Additionally, CAs also catalyse numerous other reactions, such as the conversion of cyanate to carbamic acid, cyanamide to urea, sulphonyl chlorides to sulphonic acids, aldehydes to alcohols^5^. The CA superfamily is ubiquitously distributed in all living organisms and grouped into eight CA classes indicated with the Greek letters (α, β, γ, δ, ζ, η, θ, and ι). Their distribution is very variegated, being found in complex organisms (plants and animals) as well as simpler ones (bacteria and archaea). The genome of mammals encodes only for the α-CA class, of which 15 isoforms have been identified. Besides, the α-CAs, differently from most other CA-classes, possess an esterase activity, too. The CA-catalysed reaction is involved in numerous physiological and pathological processes, including respiration and transport of CO_2_ and bicarbonate between metabolising tissues and lungs; pH and CO_2_ homeostasis; electrolyte secretion in various tissues and organs; biosynthetic reactions (such as gluconeogenesis, lipogenesis, and ureagenesis); bone resorption; calcification; tumorigenicity and virulence of multiple pathogens^5^ .Interestingly, despite the enormous steps that have been made over the last decades to understand the molecular machinery associated with CAs, the study of this superfamily of enzymes is still ongoing^5^.

The putative role of CA in the Covid-19 has arisen from the analogy between Covid-19 and the high-altitude pulmonary oedema (HAPE)^2^. Covid-19 and HAPE share these common phenomena: a decreased ratio of arterial oxygen, partial pressure to fractional inspired oxygen, and a reduction of the carbon dioxide levels; increased tachypnoea and fibrinogen levels/fibrin formation; the presence of hypoxia, lungs with ground glass opacities when analysed through chest computed tomography **(**CT), patchy infiltrates on chest X-ray radiography, alveolar compromise, and, finally, acute respiratory distress syndrome (ARDS) development in severe disease^2^. In Covid-19 patients, partial pressure of CO_2_ (pCO_2_) does not relevantly rise, if not at the latest, in most critical stages. Furthermore, the ratio of the arterial pO_2_ (PaO_2_) and the fraction (percent) of inspired oxygen that the patient is receiving (FiO_2_), which is usually high in ARDS, is conversely low in Covid-19 (or in HAPE) until critical respiratory insufficiency occurs^6^. As aforementioned, a systemic hypoxic state has been observed in up to 80% of intensive care unit (ICU) patients with regular pulmonary tissue compliance^6^.

The heterodimeric hypoxia-inducible factor 1 (HIF-1), is formed by two subunits HIF-1α and HIF-1β^7^. HIF-1α is O_2_-regulated and its expression rises progressively in hypoxic condition. It has pro-inflammatory effect via regulation of high level of IFNI, which is produced by cytokines production, such as interleukin-6 (IL-6), tumour necrosis factor-alpha (TNF-α), and signal transducer and activator of transcription 3 (STAT3) pathway^7^. The suppression of HIF-1 transcription or inhibition of its activity could reduce the inflammation caused by Covid-19 triggered in involved organs such as lungs. Interesting, the binding of the complex HIF-1α/HIF-1β to hypoxia-response elements (HREs) results in transcription of genes essential for an adaptive response against hypoxia, such as glycolytic enzymes, glucose transporters, erythropoietin, and angiogenic factor vascular endothelial growth factor (VEGF)^7^. These molecules enable the cell to survive hypoxic stress by increasing oxygen delivery through angiogenesis and inducing the anaerobic glycolysis. Moreover, HIF-1α expressed under hypoxic conditions, activates the expression of CA IX, which has a role in pH regulation, as well as influences the glucose transporter-1 (GLUT-1), and the VEGF that is involved in the angiogenesis^8^. Interesting, ACE1 and ACE2 form the oxygen-sensitive renin–angiotensin system (RAS)^9^. In normoxia, RAS is regulated by the dynamic equilibrium between the expression of ACE1 and ACE2. However, under chronic hypoxia (2% O_2_ for 12 days), ACE1 is upregulated by the HIF-1 in human pulmonary artery smooth muscle cells (hPASMCs), while the expression of ACE2 is markedly decreased^9^. Zolfaghari Emameh et al. combined system biology and bioinformatic approaches, defining the role of co-expression of ACE2, neprilysin, or membrane metalloendopeptidase (MME), and CAs in the pathogenesis of SARS-CoV-2^10^. The results revealed that ACE2, MME, and CAs significantly contribute to RAS in the pathogenesis of SARS-CoV-2. Dysregulation in these biomolecules can increase CO_2_ concentration in the blood, provoke respiratory acidosis, pulmonary oedema, and heart and renal failures^10^. Therefore, the system ACE2-neprilysin-CA could be a critical factor of the pathogenesis of SARS-CoV-2 and may stimulate researchers in finding better therapeutic strategies to contrast Covid-19^10^.

Considering these literature data, in this article, we report a study on patients affected by Covid-19 who were subjected to oximetry readings in order to determine their pO_2_ and to blood tests for measuring pCO_2_, pH, and bicarbonate concentration, as well as the total blood CA activity. We hypothesise that patients affected by Covid-19 could show an acid–base status influenced probably by the CA activity, offering the opportunity to use CA inhibitors (CAIs) as adjunctive pharmacological treatment for Covid-19. The rationale for our proposal is that the clinically employed CAI, acetazolamide, is also used to treat HEPA^2^ as this drug reduces hypoxic pulmonary vasoconstriction and improve minute ventilation as well as the expired vital capacity.

## 2. Materials and methods

### 2.1. Selection of Covid-19 and non-Covid-19 patients

The category of patients in this preliminary study is designed with acute Covid-19, post-Covid-19, and non-Covid-19 patients. Patients fell into the following Covid-19 categories: acute Covid-19: symptoms of Covid-19 for up to 4 weeks following the onset of illness; post-Covid-19: symptoms that develop during or after Covid-19 continue for ≥12 weeks, not explained by an alternative diagnosis. Non-Covid-19: the patients do not have any Covid-19 symptoms. The study is represented by 31 acute Covid-19 patients, 47 post-Covid-19 patients, and 31 non-Covid-19 patients. All patients were age ≥ 18 years old. Acute and post-Covid-19 patients were presented to the Pamukkale University Hospital with hypoxia (oxygen saturation (SpO_2_) <90%)) and without resolution (SpO_2_ >93%) from October and February (early stages of the Covid-19 outbreak). These patients were not supplemented with oxygen and were capable of self-proning.

This study is licenced by the Republic of Turkey Ministry of Health with a permission account of 2020-12-22T22_54_47 on 25 December 2020. The research ethics committee's function and purpose are to ensure that the research that will take place follows the relevant ethical standards by Pamukkale University Ethical Committee with number 10.150.1.90/106832 in 05 January 2021.

### 2.2. Diagnosis of Covid-19 patients

All considered patients were diagnosed as Covid-19 with either real-time reverse transcriptase-polymerase chain reaction (RRT-PCR) of respiratory secretions obtained nasopharyngeal- or oropharyngeal swab and endotracheal aspirate, or highly suspicious radiological findings. The chest CT was carried out using a dual slice scanner (Mx8000, Philips Medical Systems, Cleveland, OH).

### 2.3. Oximetry readings to determine the pO_2_

pO_2_ was measured through standard finger oximeters (Covidien Oximax, Covidien, Minneapolis, MN). Hypoxaemia was defined as a SpO_2_<90%.

### 2.4. pH, pCO_2_, and HCO_3_^–^ determination

pH, pCO_2_, and HCO_3_^–^ values were determined by Medica Easystat blood gas analyser (Bedford, MA).

### 2.5. Vital signs of patients

Vital signs were obtained from the cardiac monitor (Philips IntelliVue, Philips, Cleveland, OH) in real-time.

### 2.6. Measurement of CA activity

CA activity was investigated in the blood of Covid-19 patients in Ankara Yildirim Beyazit University Medical Biology research laboratory with 29 non-Covid-19 patients, 29 acute Covid-19, and 47 post-Covid-19 patients. The enzyme activity was measured by the hydration of CO_2_ according to the method of Wilbur and Anderson^11^, which is based on the determination of the time required for the pH of solution decreasing from 10.0 to 7.4 due to the hydration of CO_2_. Assays were performed at least twice on each lysate, and the mean value was determined to the formula: EU=(to – tc)/tc^12^.

## 3. Results and discussion

### 3.1. Lung computed tomography and hypoxia

**Figure 1.**
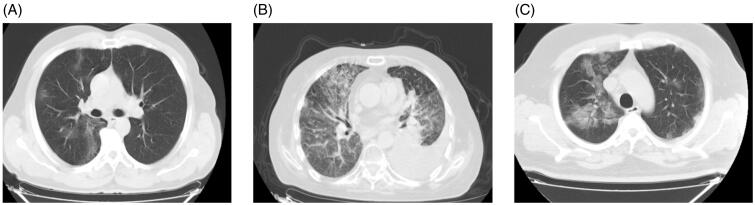
Chest CT images in COVID-19 patients at different stage of the disease (panels A–C). (A) An axial CT image obtained without intravenous contrast in a 62-year-old male shows bilateral peripheral focal ground-glass opacities (GGOs) with a rounded morphology; (B) an axial CT image obtained without intravenous contrast in a 59-year-old female shows consolidative opacities and pleural effusion on the left and ground-glass opacities including interstitial thickening on the right side; (C) an axial CT image obtained without intravenous contrast in a 29-year-old male patient demonstrates bilateral mixed ground-glass opacities and central consolidated areas of the lung.

We determined the chest CT images of patients at different Covid-19 stages. Figure 1 shows lung CTs (panels A–C). Jin et al.^13^ described the chest CT findings of Covid-19 in five temporal stages as ultra-early, early, rapid progression, consolidation, and dissipation stages. During the ultra-early stage (asymptomatic, 1–2 weeks after exposure), CT may show single or multiple focal GGO, patchy consolidative opacities, pulmonary nodules encircled by GGO, and air bronchograms. In the early stage (early symptomatic presentation, 54% of their cases), CT findings include single or multiple GGOs, or GGO combined with an interlobular septal thickening. In the rapid progression stage (days 3–7 of symptomatic presentation), CT findings include large, light consolidative opacities and air bronchograms. During the consolidation stage (second week of symptomatic presentation), reductions in density and size of the consolidative opacities may be seen. About 2–3 weeks after the onset, CT may show dispersed patchy consolidative opacities, reticular opacities (referred to as “strip-like opacities”), bronchial wall thickening, and interlobular septal thickening^14^.

It is essential to indicate that ground-glass opacities (GGOs) are not specific to Covid-19 and can be seen in many different pathologies, such as congestive heart failure, inflammatory interstitial lung diseases, diffuse alveolar haemorrhage (bleeding into the lungs' airspaces), and viral pneumonia determined by respiratory syncytial virus (RSV), cytomegalovirus, herpes simplex virus, and other CoV^15^. The scientific literature revealed that the patients with Covid-19 often develop respiratory failure 8–14 days after symptom onset, with a high respiratory rate^16^^,^^17^. Wilkerson et al.^18^ reported that patients with SARS-CoV-2 infection develop a degree of hypoxia that is proportional to the patient's symptoms. This hypoxia has been called “silent or apathetic hypoxia” and may be considered as a clinical sign to look for determining if the patients are at increased risk of sudden decompensation. Another study carried out on symptomatic individuals reported the sickness after a presymptomatic phase with no symptoms. Approximately, 80% of these people exhibit a “mild” disease course, whereas the other 20% progress to severe and critical stages associated with pneumonia, ARDS and respiratory failure, septic shock and multiorgan failure^19^. All these conditions complicate patients’ breathing and induce hypoxic conditions, which lead to extrapulmonary pathologies in vital organs, such as the brain, heart, kidneys, and others^19^. Systemic inflammation and hypoxia predispose people to being immobile and to gain weight^20^. Besides, laboratory data show a relevantly lower haemoglobin level and higher ferritin levels in non-surviving Covid-19 patients compared to survivors^6^. It has been seen that cell iron overload is tolerated up to a threshold, in patients with silent hypoxia (Covid-19 patients in the first phase)^6^. The increasing ferroptosis-linked multi-organ oxidative stress can precipitate the inflammatory/immune over-response (the so-called cytokine storm) in later and most critical stages. During profound hypoxia, tissue injury is caused by mitochondrial degeneration^6^. In the absence of sufficient oxygen supply, anaerobic glycolysis takes place with consequent repercussions in mitochondrial metabolism. Cell necrosis and altered apoptosis follow due to abnormal mitophagy; this kind of histotoxic hypoxia somehow mimics the tissue injuries caused by the pathophysiology of chronic cyanide/lead-poisoned haemoglobin^6^. It has been suggested that home monitoring with pulse oximetry devices provides earlier recognition of patients requiring medical evaluation and supportive care^18^.

### 3.2. pO2 and pCO_2_ evaluation

**Figure 2.**
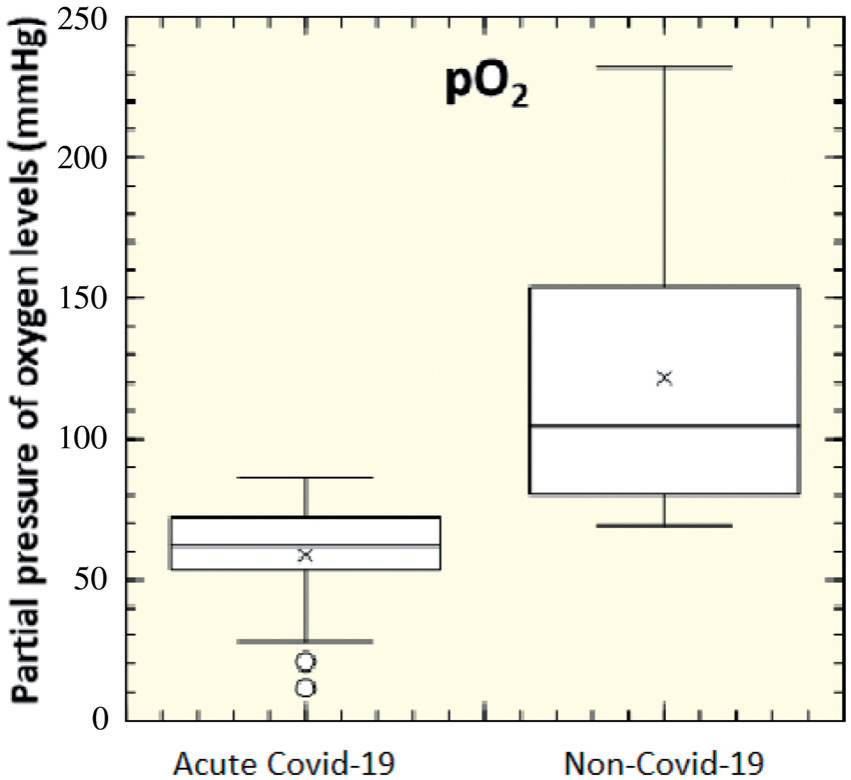
Partial pressure of oxygen levels of 31 acute-Covid-19 patients and 31 non-Covid-19 patients (*p*<.05).

Oximetry readings provide data about a patient's oxygenation status. The pO_2_ values of the 31 patients with Covid-19 have been reported in Figure 2. Non-Covid-19 patients were used as control.

As shown in Figure 2, the first observation was a significative change in pO_2_ (*p*<.05). Non-Covid-19 patients showed a pO_2_ ranging from 80 to 230 mmHg, while in Covid-19 patients it was significantly reduced up to 20 mmHg. pO_2_ was determined before proning, after applying supplemental oxygen, and after 5 min of proning without a change in inspired oxygen. The secondary outcome was the rate of patients who were prone but then required intubation within 24 h of presentation to the ED. A patient was deemed to have failed proning if they showed respiratory failure defined as persistent pO_2_<90% in the setting of unresolved or worsening tachypnoea with either accessory muscle use, altered mental status, or hypercarbia on blood gas. Interesting, both Covid-19 and HAPE exhibit decreased arterial oxygen partial pressure to fractional inspired oxygen (pO_2_:FiO_2_ ratio) with attendant hypoxia and tachypnoea.

**Figure 3.**
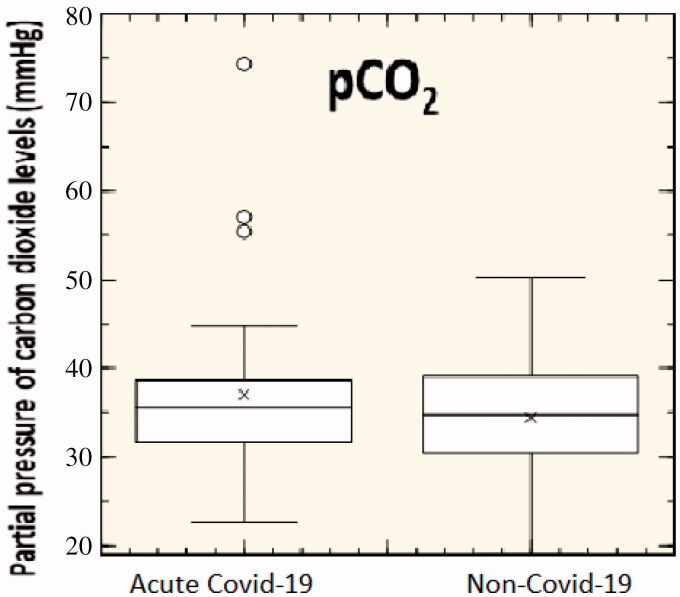
Partial pressure of carbon dioxide levels of 31 acute-Covid-19 patients and 31 non-Covid-19 patients (*p*>.05).

Generally, under normal physiologic conditions, the value of pCO_2_ ranges between 35 and 45 mmHg within arterial or venous blood. From Figure 3, it seems that blood pCO_2_ of Covid-19 and non-Covid-19 patients are rather different (*p*>.05). Intriguing, in a recent article describing 138 hospitalised cases, the median pCO_2_ level was 34 mmHg (interquartile range: 30–38; normal range: 35–48)^21^. Here, we stress that initial exposure to hypoxia at high altitudes leads to an immediate increase in ventilation that blows off large quantities of CO_2_, producing hypocapnia. Furthermore, blood gases of non-acclimatised mountaineers with severe illness were accompanied by a significant decrease in arterial oxygen due to an increase in alveolar–arterial oxygen difference. However, herein arterial pCO_2_ did not change significantly^21^. One of the essential requirements of the body is to eliminate CO_2_. The large but highly variable amount of CO_2_ produced within muscle cells has to leave the body finally via ventilation of the alveolar space. To get there, diffusion of CO_2_ has to occur from the intracellular space of muscles into the convective transport medium blood, and diffusion out of the blood has to take place into the lung gas space across the alveolocapillary barrier^22^. Covid-19 infection, damaging lung cells, impairs gas transfer provoking severe hypoxia, which is usually the cause of death when it occurs. Besides, in a substantial number of patients, adequate arterial oxygenation cannot be achieved with supplemental oxygen alone^23^.

### 3.3. Determination of CA activity, pH, and bicarbonate

**Figure 4.**
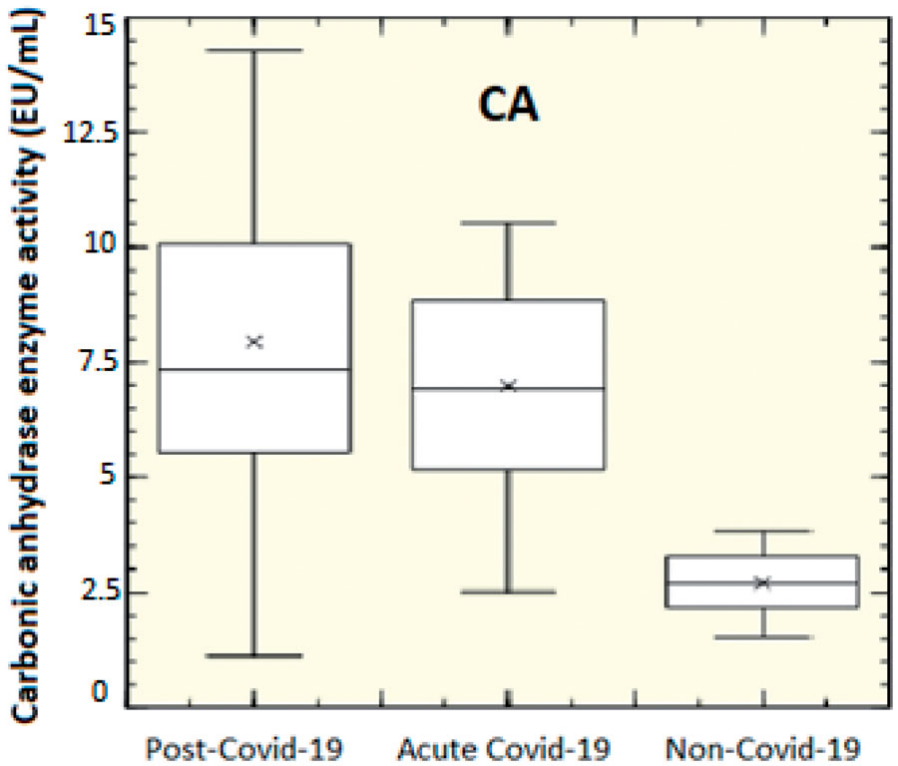
CA enzyme activity in the blood of 47 post-Covid-19 patients (after the infection, symptoms continue for ≥12 weeks), 29 acute Covid-19 patients (symptoms of Covid-19 for up to 4 weeks following the onset of illness), and 29 non-Covid-19 patients (*p*<.05).

CO_2_ in the body is present in three different forms: dissolved, bound as bicarbonate, or carbamate. The kinetics of the interchange between states become critically important and is regulated by the action of CAs. The products of their catalysed reaction (CO_2_, HCO_3_^−^, and H^+^) are required for a variety of metabolic and cellular functions. It is well established that an intraerythrocytic CA is essential for the efficient transport of CO_2_ by the blood^22^. Here, the blood CA activity was determined in recovered Covid-19 patients (recovered to the hospital), COVID-19 active patients (those not recovered but with active infection), and non-Covid-19 patients, used as control (Figure 4).

**Figure 5.**
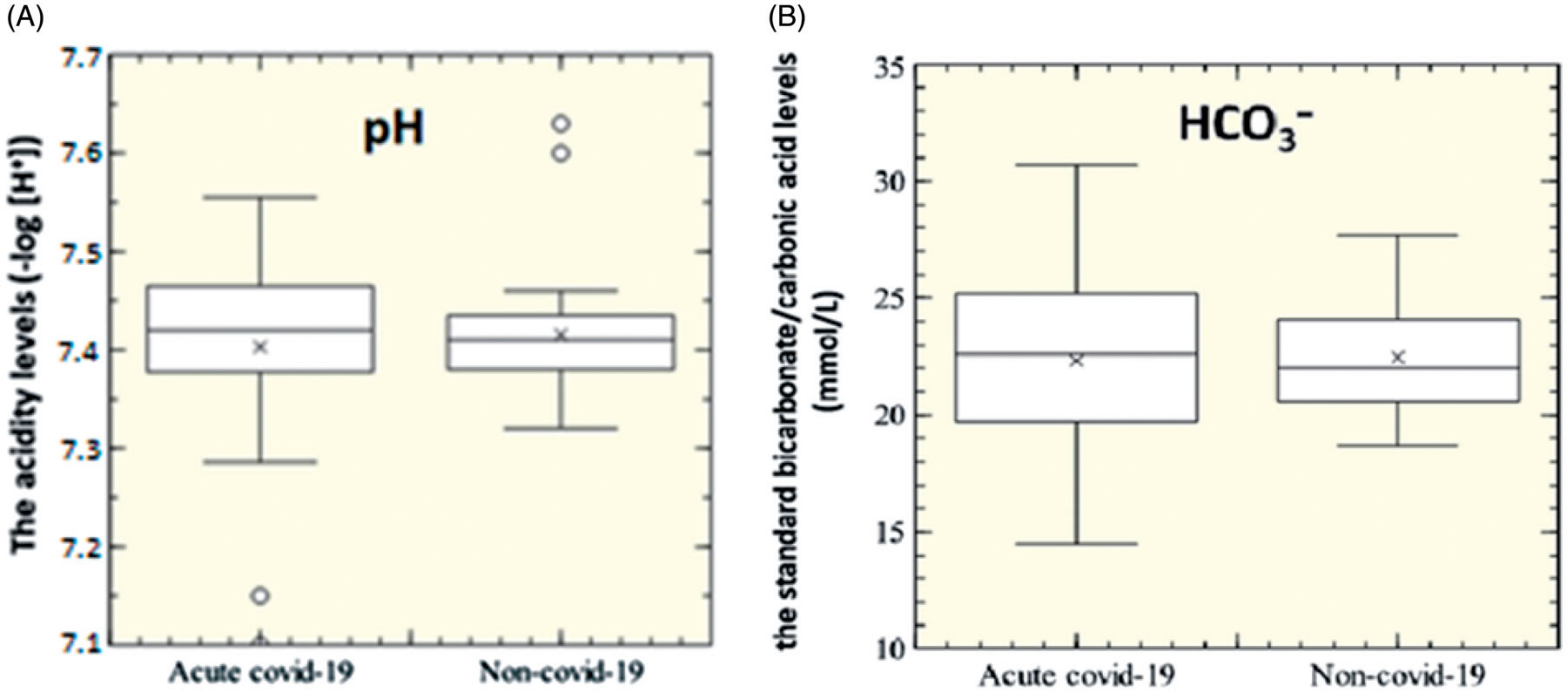
pH and bicarbonate blood tests of 31 acute-Covid-19 patients and 31 non-Covid-19 patients. (A) The acidity levels; (B) the standard bicarbonate/carbonic acid levels (*p*>.05).

As shown in Figure 4, the level of CA activity in the blood of the Covid-19 patients was significantly increased (*p*<.05). In post-Covid-19 patients, the CA activity resulted higher than acute Covid-19 patients. We hypothesise here that the higher CA activity measured in Covid-19 patients could be a body response to excessive carbon dioxide, responsible of the hypercapnia, hypercarbia, or hypercapnia. This may suggest that the use of CAIs could be important in the treatment of Covid-19 patients with serious respiratory conditions, as this would reduce the level of blood CA activity. Interestingly, the increased enzyme activity is supported by the results obtained measuring the acidity (pH) and the bicarbonate concentration in the blood of patients affected by Covid-19 (Figure 5).

Figure 5 shows a mild increase of pH (panel A) and bicarbonate (panel B) in Covid-19 patients. The acute Covid-19 mean value is 7.40; the non-Covid-19 mean value is 7.42. The increase in pH could be caused by a higher concentration of HCO_3_^−^ relative to a low pCO_2_. CO_2_ is released from carboxyhaemoglobin and dissolved in blood quickly once the concentration of CO_2_ gas was switched from 10% to 5%, more CO_2_ must have been produced progressively by CA so that a relatively high concentration of HCO_3_^−^ was kept until the pCO_2_ in the collected blood was measured^24^. Changes in CA activity can affect pH in red blood cells (RBCs) due to a transient imbalance between CO_2_ and HCO_3_^−^ concentrations, which could change the ability of Hb to bind oxygen and briefly shift the oxyhaemoglobin dissociation curve to the right or left for a short time. Thus, CA has the potential of being used to modify the affinity of Hb for oxygen in the treatment of heart and brain ischaemia^24^.

Several drugs, such as chloroquine and hydroxychloroquine, were proposed as possible treatments of Covid-19, as they may increase the haemoglobin production and its availability for oxygen binding^25^. The use of acetazolamide that causes hyperventilation with associated increased levels of oxygen and decreased levels of carbon dioxide in the blood was also proposed^25^. Acetazolamide potently inhibits CAs and has been shown to cause an increase in the cytosolic pH^26^. Furthermore, over 70% of patients with Covid-19 had elevated lactate dehydrogenase levels, which may be connected to hypoxia, too^21^. Acetazolamide has pharmacologic effects also in delaying plasma lactate appearance with no impact on the ventilatory threshold^21^. Acetazolamide has a myriad of effects on different organ systems^21^. Acetazolamide inhibits the re-absorption of ions, and hence, by increasing the blood level of bicarbonate, the blood becomes acidic. This results in compensatory hyperventilation (Kussmaul respiration) with increased blood oxygen levels and decreased blood carbon dioxide levels. As a result, acetazolamide treatment may improve respiratory conditions related to Covid-19 by a mechanism consistent with the various previously considered treatments^25^. It potently reduces hypoxic pulmonary vasoconstriction, improves minute ventilation, and expires vital capacity seen in climbers taking acetazolamide^21^.

## 4. Conclusions

Patients affected by Covid-19 could show a dysregulated acid–base status probably influenced by the CA activity, which is highly increased in patients affected by Covid-19 infection. This is also supported by a slightly increased blood pH level, higher concentration of HCO_3_^−^ and relatively low pCO_2_. Several treatments, such as tocilizumab, an anti-IL-6 receptor monoclonal antibody, and dexamethasone, a glucocorticoid medication for rheumatic problems, have been used to treat Covid-19 patients^27^^,^^28^. The first improves patients' survival, shortening the time to discharge and reducing the need for a mechanical ventilator. The second is a cheap and widely available steroidal drug, which is considered indispensable for many Covid-19 patients. Interestingly, Simko et al.^29^ demonstrated that dexamethasone decreases CA IX expression via the HIF-1α-dependent mechanism in 2D and 3D cancer cell models. It was suggested that the effect of dexamethasone on CA expression under hypoxic conditions is predominantly mediated by impaired transcriptional activity and decreased protein level of the main hypoxic transcription factor HIF-1α^29^. Thus, classical CAIs (such as acetazolamide, methazolamide, etc.), decreasing the enzyme activity, could be considered an adjunctive pharmacological treatment for Covid-19. CAIs might represent a rational alternative approach to relieve patients of Covid-19 disorder, especially for the similarity that this infection shows with HEPA, but additional studies are needed in order to understand this relevant increase of CA activity in Covid-19 patients.
